# Sleep and cognition in aging dogs. A polysomnographic study

**DOI:** 10.3389/fvets.2023.1151266

**Published:** 2023-04-28

**Authors:** Alejandra Mondino, Magaly Catanzariti, Diego Martin Mateos, Michael Khan, Claire Ludwig, Anna Kis, Margaret E. Gruen, Natasha J. Olby

**Affiliations:** ^1^Department of Clinical Sciences, College of Veterinary Medicine, North Carolina State University, Raleigh, NC, United States; ^2^Instituto de Matemática Aplicada del Litoral, Consejo Nacional de Investigaciones Científicas y Técninas, Universidad Nacional del Litoral, Santa Fe, Argentina; ^3^Physics Department, Universidad Autónoma de Entre Ríos (UADER), Oro Verde, Entre Ríos, Argentina; ^4^Research Centre for Natural Sciences, Institute of Cognitive Neuroscience and Psychology, Budapest, Hungary

**Keywords:** NREM sleep, REM sleep, canine cognitive dysfunction syndrome, quantitative EEG, power spectrum, coherence, complexity

## Abstract

**Introduction:**

Sleep is fundamental for cognitive homeostasis, especially in senior populations since clearance of amyloid beta (key in the pathophysiology of Alzheimer's disease) occurs during sleep. Some electroencephalographic characteristics of sleep and wakefulness have been considered a hallmark of dementia. Owners of dogs with canine cognitive dysfunction syndrome (a canine analog to Alzheimer's disease) report that their dogs suffer from difficulty sleeping. The aim of this study was to quantify age-related changes in the sleep-wakefulness cycle macrostructure and electroencephalographic features in senior dogs and to correlate them with their cognitive performance.

**Methods:**

We performed polysomnographic recordings in 28 senior dogs during a 2 h afternoon nap. Percentage of time spent in wakefulness, drowsiness, NREM, and REM sleep, as well as latency to the three sleep states were calculated. Spectral power, coherence, and Lempel Ziv Complexity of the brain oscillations were estimated. Finally, cognitive performance was evaluated by means of the Canine Dementia Scale Questionnaire and a battery of cognitive tests. Correlations between age, cognitive performance and sleep-wakefulness cycle macrostructure and electroencephalographic features were calculated.

**Results:**

Dogs with higher dementia scores and with worse performance in a problem-solving task spent less time in NREM and REM sleep. Additionally, quantitative electroencephalographic analyses showed differences in dogs associated with age or cognitive performance, some of them reflecting shallower sleep in more affected dogs.

**Discussion:**

Polysomnographic recordings in dogs can detect sleep-wakefulness cycle changes associated with dementia. Further studies should evaluate polysomnography's potential clinical use to monitor the progression of canine cognitive dysfunction syndrome.

## 1. Introduction

Sleep is fundamental for cognitive homeostasis as some of its most relevant functions are memory consolidation and learning processing (1–3). Dogs, like humans and other mammals, experience age-related changes in their sleep-wakefulness cycle (4–8). In fact, owners of aging dogs report that sleeplessness is an age-related behavioral change with great impact on the owner-pet relationship (9). While these variations can be attributed to normal aging, in some cases they may possibly be due to underlying neurodegenerative processes (10, 11).

Humans with Alzheimer's disease (AD), for example, experience sleep disruptions, such as insomnia and sleep fragmentation. These signs can occur early in the course of the disease because brain regions that regulate sleep and circadian rhythms, such as the suprachiasmatic nuclei, are some of the first to be affected (10, 12, 13). Older dogs are prone to develop canine cognitive dysfunction syndrome (CCDS), a disease similar to AD (14–16). This syndrome is characterized by disorientation, memory impairment and difficulty learning, changes in social interactions and house soiling behaviors, increased anxiety, and alteration of the sleep-wakefulness cycle (11, 17–19). Specifically, owners of dogs with CCDS report that their dogs suffer from difficulty sleeping, increased pacing and vocalizations at nighttime, and/or increased sleeping at daytime (11, 20).

While sleep disturbance might be a consequence of these neurodegenerative processes, they can also contribute to the pathophysiology of the disease and to memory impairment resulting in a vicious cycle (13, 21). Clearance of amyloid beta (Aβ) occurs in the brain through the glymphatic system, and this system is primarily active during slow wave sleep (NREM sleep) (22). Therefore, sleep deprivation can increase Aβ deposition in the brain (13, 22–25). While several studies have shown that sleep is disrupted in dogs with CCDS (11, 26), this has been based on owner reports, and no study has looked at the macrostructure or at the electroencephalographic changes of sleep in these dogs. Polysomnography is the gold standard technique to objectively evaluate sleep (27) and a non-invasive technique has been developed in dogs (28). It consists of the simultaneous recording of the EEG, electrical activity of the muscles (EMG) and eye movements (EOG) and can also include the recording of the electrocardiogram (ECG) and respiratory movements.

In addition to the importance of an adequate amount of time dedicated to sleep, the electroencephalographic characteristics of sleep are also associated with Aβ clearance; for example, the reduction of slow oscillations (delta) power is strongly correlated with Aβ deposition in the brain (25). In fact, studies in humans using quantitative electroencephalographic analyses (qEEG) (27) have shown electroencephalographic signatures of dementia during wakefulness and sleep states (29–33). One of the most widely employed qEEG analyses is spectral power, which provides the relative contribution of the frequency components of a signal and reflects the local degree of synchronization of the extracellular potential (34). Spectral power is calculated by means of the fast Fourier transform, and the details of its calculation can be found elsewhere (27). Usually, the frequency spectrum is divided into discrete ranges (bands); in dogs, these are delta (1–4 Hz), theta (4–8 Hz), alpha (8–12 Hz), sigma (12–16 Hz), beta (16–30 Hz), and gamma (30–45 Hz) (21). Each band is associated with different behavioral or consciousness states, for example, wakefulness is characterized by higher power in faster oscillations (alpha, beta, and gamma), while NREM sleep or anesthetic states are characterized by higher power in delta oscillations (27, 35, 36). In humans with AD, an increase in delta power and a reduction in the frequency of the peak alpha power has been demonstrated during resting state (37).

Another qEEG analysis frequently used is the spectral coherence, which measures the degree of functional coupling between two cortical areas. Two brain oscillations are completely coherent at a specific frequency if their phase difference and the relationship between the amplitudes at that frequency are constant (27, 38). Coherence can be calculated between two areas within the same brain hemisphere (intrahemispheric) or between areas on the right and left hemispheres (interhemispheric) We have previously shown that, comparable to people with AD (39, 40), during wakefulness, dogs with presumptive diagnosis of CCDS show a reduction of interhemispheric coherence in the high frequency bands (41).

Both power and coherence are measures of linear dynamics, but the complex EEG signal cannot be explained only by them. Non-linear analyses are a more modern method to study the complexity of the system (42). One of the most used in qEEG research is Lempel Ziv Complexity, which evaluates the randomness of finite sequences based on the number of distinct patterns in a signal and the symbolic encoding (43). The LZC analysis is usually performed on the whole signal of a wider range of frequencies, without analyzing different frequency bands. This complexity has been used in the analysis of different neurophysiological signals, for example, to study the EEG signatures of anesthesia, sleep, and altered states of consciousness, as well as brain disorders such as AD or epilepsy (44–47). During wakefulness, people with AD show a reduction in Lempel Ziv Complexity (45), but we have recently found that the opposite is true for dogs with presumptive diagnosis of CCDS (41).

The aim of this study was to describe and quantify age-related changes in the sleep-wakefulness cycle macrostructure and EEG features in senior dogs and to correlate them with their cognitive performance. We predicted that cognitively impaired dogs would sleep less and would have distinct EEG features from dogs without cognitive impairment.

## 2. Methods

### 2.1. Study population

All procedures were approved by the North Carolina State University (NCSU) Institutional Animal Care and Use Committee, protocol number: 21-303. Client-owned dogs participating in the longitudinal study of neuro-aging at the North Carolina State University (NCSU), College of Veterinary Medicine were used in this study [described in our previous research (18, 48, 49)]. All owners reviewed and signed an informed consent form prior to participation. To be included, dogs had to be older than the 75% of their expected lifespan, which was calculated using the formula proposed by Greer et al. (50) that takes into consideration the dog's height and weight and can thus be applied in case of mixed-breed dogs as well. Additionally, they had to be free of comorbidities that could impede their ability to perform cognitive tests such as inability to walk or blindness.

Demographic information was collected, and physical, orthopedic, and neurological examinations were conducted for every dog. Height to the dorsal aspect of the scapula (withers) and body weight were measured in order to calculate dogs' expected lifespan. Urinalysis, biochemistry panel and complete blood cell count were also performed to rule out potential comorbidities.

### 2.2. Polysomnographic studies

**Figure 1:**
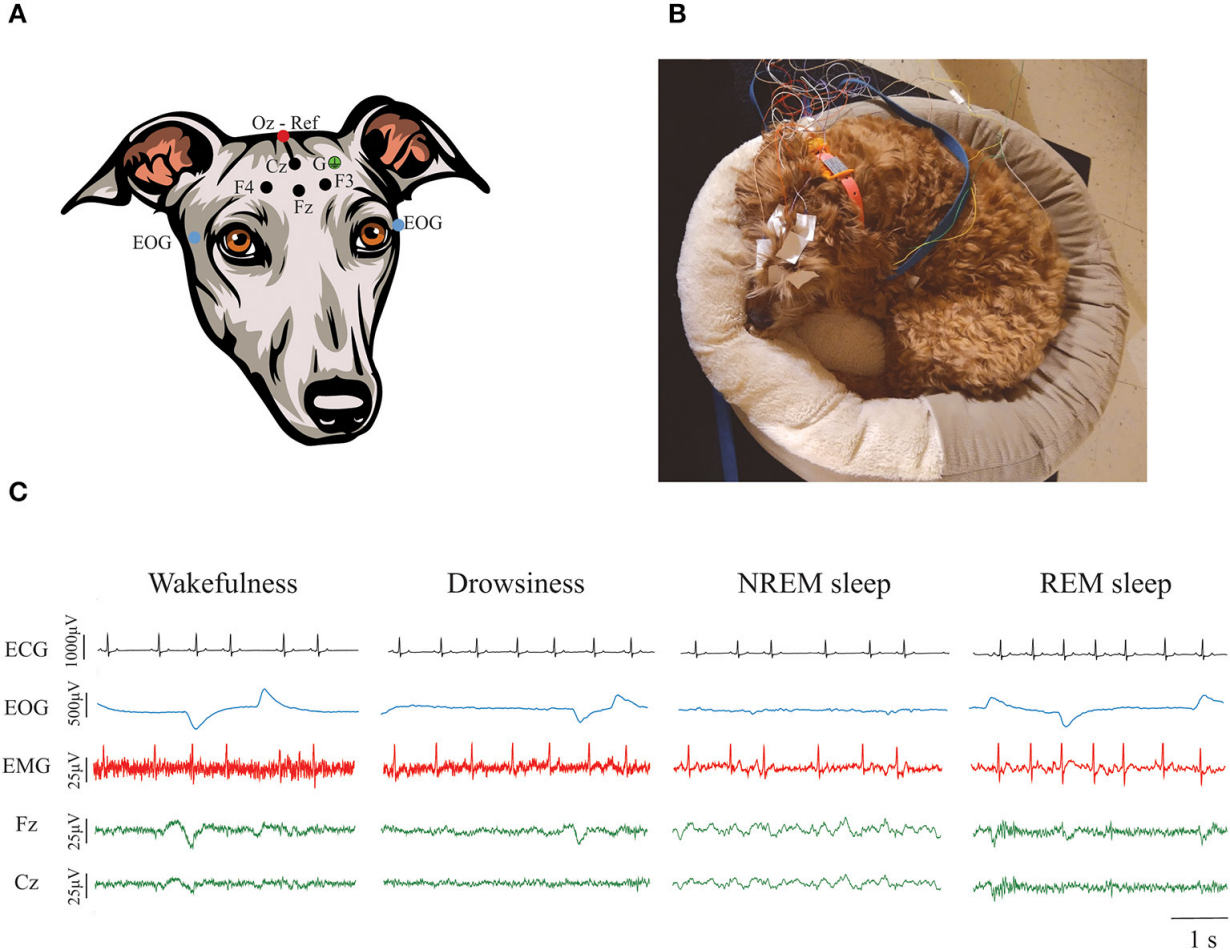
Polysomnographic recordings in dogs. **(A)** Electrode locations. Four active EEG electrodes were placed over the midline, left and right frontal areas (Fz, F3, and F4, respectively), and over the vertex (Cz). Those electrodes are represented with a black dot. A reference electrode was placed over the external occipital protuberance (Oz) and it is represented with a red dot. Bipolar EOG signal was recorded by placing two electrodes on the left and right zygomatic arch next to the lateral canthus of each eye (blue dots). A ground electrode (G) was placed over the left temporal muscles (green dot). EMG and ECG were also recorded but it is not shown in the figure. **(B)** Picture of a dog undergoing a polysomnographic recording using their own bed. **(C)** Representative ECG, EOG, EMG, and EEG signals during wakefulness, drowsiness, NREM, and REM sleep. Contamination of the ECG can be observed in the EMG signal.

Dogs underwent polysomnographic recordings with simultaneous recording of EEG, EOG, EMG, and ECG with a slight modification of the protocol described by Reicher et al. (51). As schematized in Figure 1A, we employed four active EEG electrodes, F3, F4, Fz (left, right, and midline frontal, respectively), and Cz (at the level of the vertex). The electrode location was chosen to evaluate the frontal and parietal cortex, the frontal cortex being particularly important in cognitive decline (52). These electrodes were referenced to Oz, an electrode placed over the external occipital protuberance. Bipolar EOG signal was recorded by placing two electrodes on the left and right zygomatic arch next to the lateral canthus of each eye (F7 and F8, respectively). A ground electrode was placed over the left temporal muscles. For EMG, bipolar signal was obtained by placing two electrodes over the dorsal neck muscles on the left and right side. Finally, one electrode was placed over the fifth intercostal space and referenced to the Cz electrode to record the ECG. We used gold-coated electrodes (Genuine Grass 10 mm Gold Cup, Natus Medical Inc) which were attached to the skin with SAC2 electrode cream (Cadwell Laboratories) after applying a skin preparation and electrode solution (Signa Spray, Parker Laboratories). Recordings were performed with the software Cadwell Easy II software (Cadwell Laboratories). Electrode impedance was checked to be under 20 kΩ before starting the recording. A frequency sample of 400 Hz was used. All signals were bandpass filtered between 0.53 and 70 Hz. A notch filter was also applied during acquisition to remove the 60 Hz power-line noise.

Recordings were performed in a very quiet room, with dim light and white noise reproduced through a laptop computer. Room temperature was maintained at 20°C. Owners were asked to bring their dogs for polysomnography recordings on 2 different days, an adaptation day [to avoid the “first-night-effect” (51)], and a recording day and were instructed to bring their dogs' usual bed or blankets to both recording days, so dogs felt more comfortable to sleep. Figure 1B shows an example of one dog during a polysomnography recording. During the adaptation day we performed a 30 min recording that served to acclimate the dog to the recording room and setup. On the actual testing day, the dogs had a 2-h polysomnography recording that started between 12:30 and 1:30 PM. This period of time was selected because it has been shown that dogs usually have naps around noon (5, 53, 54). For dogs who became anxious and attempted to leave the room or removed all the electrodes, we stopped the recordings even if the 2 h were not complete. Time between the adaptation and the actual recording day was never more than 2 weeks, and cognitive testing happened in between these days.

States of wakefulness, drowsiness, NREM and REM sleep were manually scored in 3 s epochs in the Spike 2 software (Cambridge Electronic Design) using criteria described previously (28). As shown in Figure 1C, wakefulness was characterized by fast frequency EEG activity, high activity in the EMG, and frequent high-amplitude eye movements. Drowsiness was defined by EEG activity similar to wakefulness, reduced but observable muscle activity in the EMG and decreased amplitude and frequency of eye movements. NREM sleep was characterized by low-frequency and high-amplitude neural oscillations, mainly within the delta frequency band. Muscle activity during NREM sleep was significantly reduced and there were no or sporadic low-amplitude eye movements. Finally, REM sleep was recognized by high frequency activity in the EEG, absence of muscle activity and frequent rapid eye movements that can also be observed as artifacts in the EEG (28). Latency to enter drowsiness, NREM and REM sleep as well as the percentage of the total recording time spent in each behavioral state were calculated. Additionally, sleep efficiency was calculated by adding the time spent in NREM and REM sleep and dividing it by the total time recorded.

### 2.3. qEEG analyses

Similar to our previous study (41), raw EEG signals from the four EEG electrodes were exported from Spike 2 to MATLAB (version 2022b; The MathWorks Inc). All artifact-free, non-transition epochs were selected for quantitative EEG analysis. Power spectrum was calculated for Fz and Cz electrode locations with a 0.25 Hz resolution between 0.5 and 50 Hz by means of *pwelch* function in MATLAB using the following parameters: 1 s sliding windows with 50% overlap. Power at F3 and F4 were not calculated since we were interested in changes in frontal and parietal cortices and analyzing three different electrodes of the same area was redundant. Total power was computed by summing the power of each 0.25 Hz bin and relative power was calculated by dividing power over total power. Interhemispheric (between F3 and F4) and intrahemispheric (between Fz and Cz) coherence were calculated using the MATLAB *mscohere* function utilizing the same parameters used for power spectrum analysis. Coherence values were then normalized using Fisher's z-transform.

We also evaluated the complexity of the EEG signal by means of Lempel-Ziv complexity for Fz and Cz. To perform the analysis, the signal was pre-processed by applying an order 5 butterworth band-pass filter between the frequencies 1 and 50 Hz. We then used the *lziv_complexity* function from the *AntroPy* package from Python (55).

### 2.4. Cognitive evaluation

We evaluated cognitive status in these dogs by using an owner-based questionnaire and a battery of cognitive tests. As in our previous studies, the questionnaire utilized was the Canine Dementia Scale (CADES), a validated clinical metrology instrument to capture behavioral changes associated with CCDS. In this questionnaire owners need to specify the frequency with which their dogs experience specific behaviors in four different domains: spatial disorientation, social interactions, sleep-wakefulness cycle and house soiling (11). CADES scores range from 0 (normal) to 95 (severely affected) and allow classification of dogs into four different categories: normal (0–7), mild cognitive impairment (MiCI, 8–23), moderate cognitive impairment (MoCI, 24–44), and severe cognitive impairment (SCI, 45–95).

Cognitive testing was also performed to objectively evaluate different domains of cognition: attention (sustained gaze test), working memory and executive control [inhibitory control and detour (cylinder) tasks]. The detailed procedure for each task has been described by our group elsewhere (18, 56). In brief, the sustained gaze task measures how long the dog holds the gaze with an experimenter who is holding a treat near their face. To evaluate working memory, two red Solo^®^ cups were placed on a mat and a small food treat was hidden under one of them (while the dog is allowed to see where it was placed). The delay (up to 2 min) between placing the treat and allowing the dog to choose one of the cups to retrieve the treat was progressively increased. The upper threshold of time that dogs were able to correctly identify (at least four out of six times) where the treat was hidden was then calculated. In the inhibitory control task, dogs were asked to retrieve a treat from a transparent plastic cylinder that is open on both ends, without touching the outside walls of cylinder. The detour task is similar but adds an additional difficulty—the dog's preferred side for retrieving the treat is now covered, and they need to enter the cylinder from the opposite side (detour) in order to obtain the treat. The outcome measure for these two tests is the percentage of correct trials (retrieving the treat without touching the outer walls of the cylinder or the detour).

### 2.5. Statistical analysis

We calculated the correlation between the sleep architecture variables (and Lempel Ziv Complexity) and age, CADES score, and performance in each of the cognitive tests by means of Spearman correlation analyses. While fractional lifespan (instead of chronological age) was employed for inclusion criteria, we used chronological age in our analyses because previous studies that have looked at sleep and qEEG analysis in dogs have reported chronological age (28, 51, 57). Statistical significance was adjusted using the Benjamini, Krieger and Yekutieli method (58) with a false discovery rate of 0.05. Adjusted *p*-value is expressed with a “q.” To evaluate the correlation between age and cognitive performance with power and coherence we did a bin to bin spearman correlation analysis and we used the procedure known as Rüger's areas (59) previously employed in polysomnographic studies in dogs (28, 57) to address the issue of multiple comparisons. These areas can be defined as a cluster of conventionally significant (*p* < 0.05) results, which are considered significant (or not) as a whole. A Rüger's area is the cluster of all the adjacent, consecutive frequency bins which contain a significant result surrounded by bins containing non-significant results. After defining these areas of significance, the number of frequency bins within the area was counted, and it was inspected to see if at least half of the *p*-values were lower than 1/2 of the conventional *p* = 0.05 significance level (i.e., below 0.025) and at least 1/3 of them were lower than 1/3 of the conventional *p* = 0.05 (i.e., 0.0167). If both requirements were satisfied, the area as a whole was considered significant.

## 3. Results

### 3.1. Demographics

Twenty-eight dogs [13.25 ± 1.57 years old (Range: 10.4–16.2)] were enrolled in this study. Their mean fractional lifespan was 1.06 ± 0.12 (Range 0.81–1.22). Seventeen were spayed females and 11 were castrated males. Seventeen were purebred dogs, and 11 were mix breed. The most represented breed was pit bull terrier with four dogs, followed by Labrador retriever with two dogs. There were also 1 dog of each of the following breeds: Australian shepherd, Basset hound, beagle, Border collie, Brittany spaniel, dachshund, German shorthaired pointer, golden retriever, German shepherd, Irish setter, and Pomeranian.

### 3.2. Polysomnography recordings

**Figure 2:**
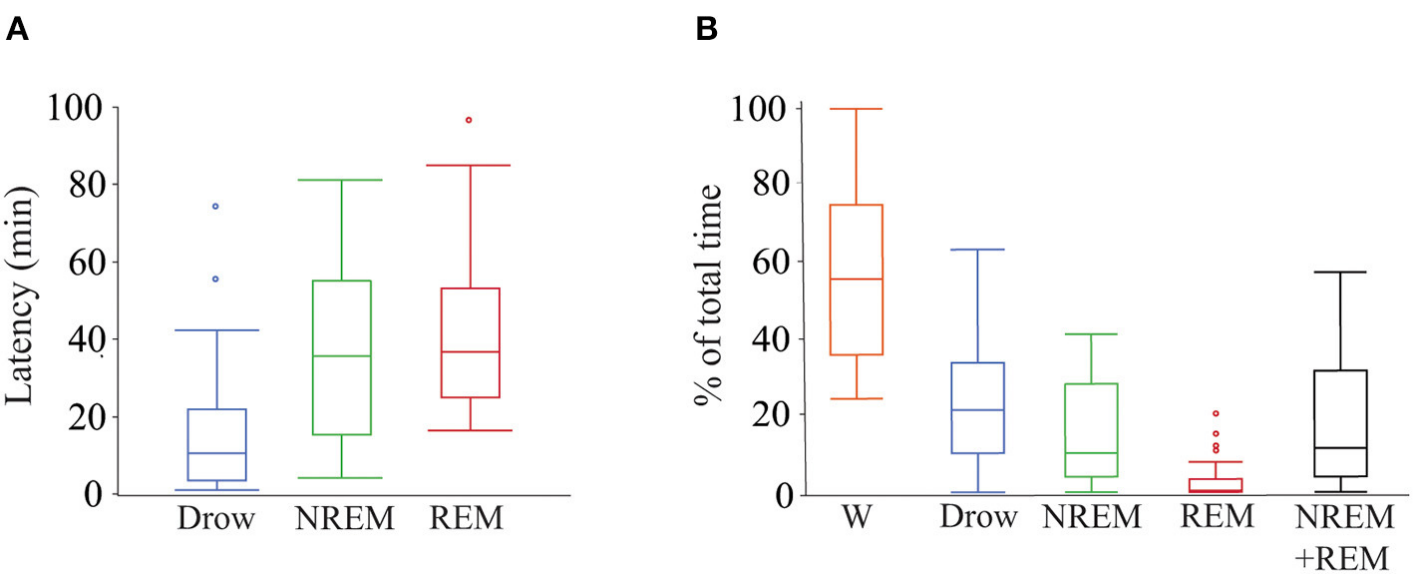
Latency to and percentage of time spent in each behavioral state during polysomnography recordings. **(A)** Box plots showing latency in minutes to drowsiness, NREM and REM sleep. Only data from dogs that entered each specific behavioral state was employed (drowsiness *n* = 26, NREM sleep *n* = 24, and REM sleep *n* = 15). **(B)** Box plots showing percentage of total time spent in each behavioral state and the sleep efficiency (sum of time in NREM and REM sleep over total time). All dogs (*n* = 28) were used. Box plots represent the median (central line of the box), the 25 and 75th percentile (lower and upper line of the box). The whiskers represent 1.5 times the interquartile range (1.5^*^IQR) and outliers are shown with a circle. W, wakefulness; Drow, drowsiness; NREM + REM, sleep efficiency.

Of the 28 dogs, 17 completed 2 h of recording. Of the remaining 9, five completed at least 1.5 h of recording and the minimum recording time was 1.01 h. Twenty-six dogs entered drowsiness, 24 into NREM sleep and 15 into REM sleep. Percentage of the total recorded time spent in each behavioral state, sleep efficiency and latency to drowsiness, NREM sleep, and REM sleep are shown in Figure 2.

### 3.3. Cognitive evaluation

Median score in the CADES questionnaire was 18 (Range 0–70). According to their score, eight dogs (28.5%) were classified as normal, eight dogs (28.5%) with mild, four dogs (14.3%) with moderate, and eight dogs (28.5%) with severe cognitive impairment. Every dog completed the sustained gaze, and inhibitory control tasks. Memory task was not performed in three of the dogs because they failed to pass warm-ups (required as a criterion for learning; they did not remember where the treat was even immediately after showing them the location, therefore, these dogs scored 0 for memory) and one dog did not complete the detour task because they lost interest and would no longer engage with the task. For this dog, detour task data were excluded from the analysis. The median sustained gaze time was 17.0 s (range 1.3–60), median memory threshold was 40 s (range 0–120), median percentage of correct trials in inhibitory control was 100 (range 13–100%) and in detour 50 (range 0–100%).

### 3.4. Correlation between sleep macrostructure and age and cognitive status

**Figure 3:**
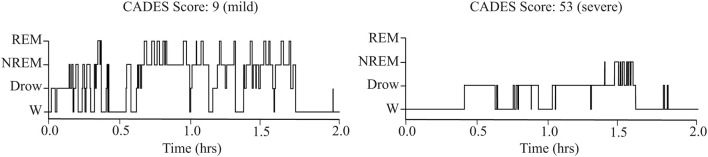
Representative hypnograms showing the difference in sleep architecture between a dog with mild **(left)** and severe **(right)** cognitive impairment based on CADES score. W, wakefulness; Drow, Drowsiness.

As shown in Table 1, older dogs tended to spend more time awake and less time in NREM sleep. However, these correlations did not reach statistical significance after correcting for multiple comparisons. There was also a relationship between cognitive performance and sleep parameters: the CADES score was negatively correlated with time spent in NREM and REM sleep. Dogs with higher CADES scores, also tended to have a higher latency to enter into NREM sleep. Figure 3 shows the hypnogram of two representative dogs, one with a low CADES score (classified as with mild cognitive impairment) and one with a high CADES score (classified as with severe cognitive impairment). This figure clearly illustrates how dogs with higher CADES scores spend less time sleeping. Additionally, performance in the detour task was negatively correlated with time spent in wakefulness and positively correlated with time spent in NREM and REM sleep. Dogs with worse performance also tended to have longer NREM sleep latency. We did not find any association between sleep macrostructure and performance in any of the other cognitive tests.

**Table 1:** Correlation between sleep macrostructure and age and cognitive function.

**Variable**	**By variable**	**Spearman ρ**	**Raw *p*-value**	***q*-value**
Age	Latency to Drow.	0.229	0.260	0.439
Age	Latency to NREM	0.440	0.031	0.116
Age	Latency to REM	0.091	0.747	0.783
Age	Time in W (%)	0.408	0.020^*^	0.116
Age	Time in Drow. (%)	−0.373	0.051	0.164
Age	Time in NREM (%)	−0.441	0.019^*^	0.092
Age	Time in REM (%)	−0.305	0.114	0.295
CADES	Latency to Drow.	0.210	0.304	0.472
CADES	Latency to NREM	0.436	0.033^*^	0.116
CADES	Latency to REM	0.011	0.969	0.897
CADES	Time in W (%)	0.454	0.015^*^	0.085
CADES	Time in Drow. (%)	−0.267	0.169	0.331
CADES	Time in NREM (%)	−0.554	<0.001^*^	**0.020** ^*^
CADES	Time in REM (%)	−0.553	0.002^*^	**0.020** ^*^
Memory	Latency to Drow.	−0.197	0.357	0.496
Memory	Latency to NREM	−0.319	0.147	0.331
Memory	Latency to REM	−0.258	0.395	0.529
Memory	Time in W (%)	−0.361	0.077	0.229
Memory	Time in Drow. (%)	0.272	0.189	0.349
Memory	Time in NREM (%)	0.327	0.110	0.295
Memory	Time in REM (%)	0.283	0.170	0.331
Inhibitory control	Latency to Drow.	−0.212	0.298	0.472
Inhibitory control	Latency to NREM	−0.148	0.489	0.613
Inhibitory control	Latency to REM	0.123	0.661	0.755
Inhibitory control	Time in W (%)	0.014	0.942	0.893
Inhibitory control	Time in Drow. (%)	−0.055	0.781	0.798
Inhibitory control	Time in NREM (%)	0.079	0.691	0.767
Inhibitory control	Time in REM (%)	0.111	0.572	0.674
Detour	Latency to Drow.	−0.193	0.355	0.496
Detour	Latency to NREM	−0.525	0.010^*^	0.065
Detour	Latency to REM	−0.184	0.529	0.642
Detour	Time in W (%)	−0.556	0.003^*^	**0.020** ^*^
Detour	Time in Drow. (%)	0.149	0.457	0.592
Detour	Time in NREM (%)	0.683	<0.001^*^	**0.019** ^*^
Detour	Time in REM (%)	0.596	0.001^*^	**0.019** ^*^
Eye gaze	Latency to Drow.	−0.019	0.926	0.893
Eye gaze	Latency to NREM	−0.305	0.147	0.331
Eye gaze	Latency to REM	−0.343	0.211	0.372
Eye gaze	Time in W (%)	0.193	0.324	0.484
Eye gaze	Time in Drow. (%)	0.041	0.837	0.833
Eye gaze	Time in NREM (%)	−0.071	0.718	0.774
Eye gaze	Time in REM (%)	−0.273	0.160	0.331

Asterisks and bold text indicate significant differences.

q-values represent the corrected p-values using the false discovery rate Benjamini, Krieger, and Yekutieli procedure.

W, Wakefulness; Drow, Drowsiness.

### 3.5. Associations between electroencephalographic features, age, and cognitive status

**Figure 4:**
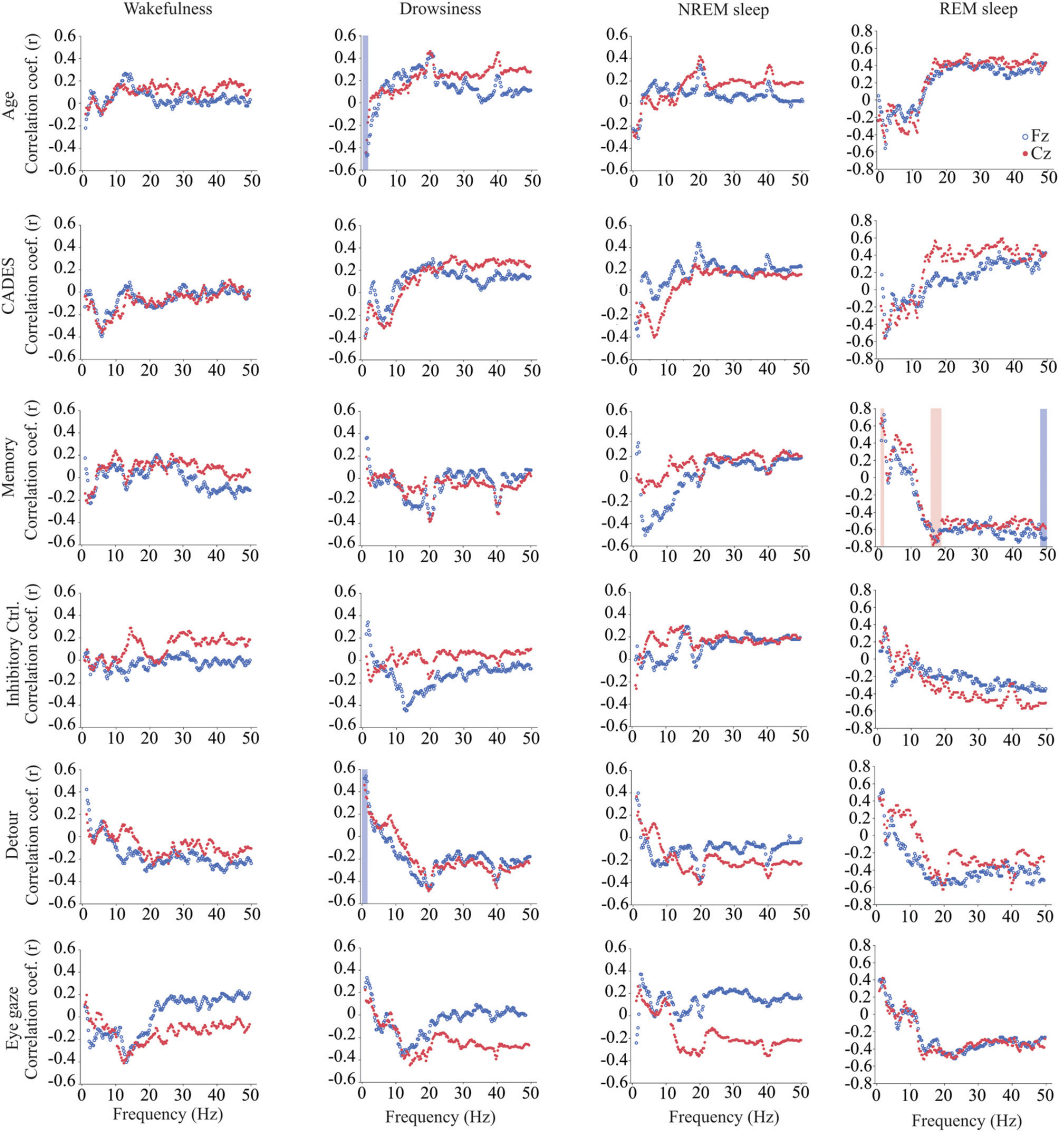
Correlations between wakefulness, EEG power and age, CADES score, or performance in cognitive tests. Correlation coefficients for the EEG channels Fz (blue open circle) and Cz (red solid circle) are shown for each frequency bin. Frequencies with significant correlations are indicated with a blue (for Fz) or red (for Cz) shadow box. The analysis was performed only for the dogs that entered each specific behavioral state (wakefulness *n* = 28, drowsiness *n* = 26, and NREM sleep *n* =24).

**Figure 5:**
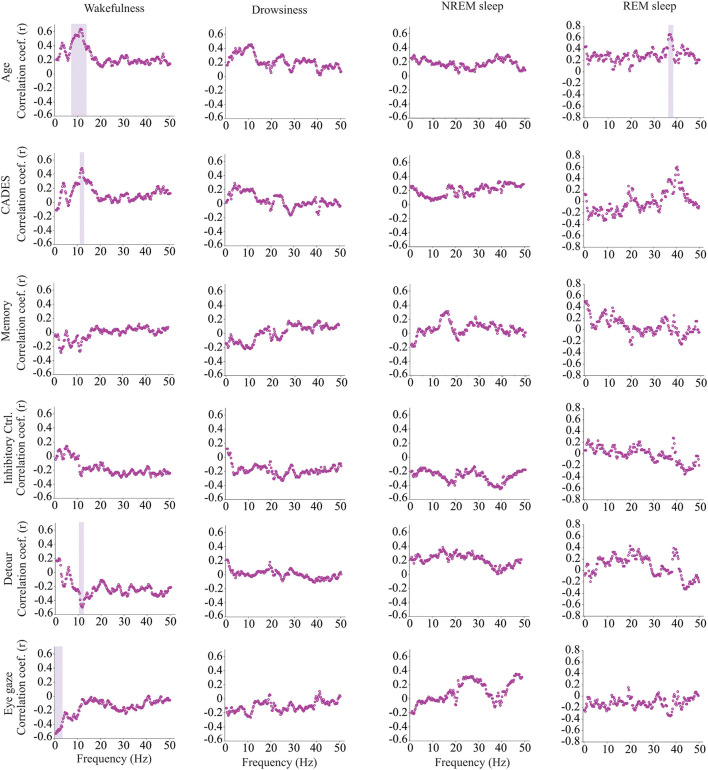
Correlations between EEG interhemispheric (F3–F4) coherence and age, CADES score, or performance in cognitive tests. Spearman correlation coefficients are shown for each frequency bin. Frequencies with significant correlations are indicated with a purple shadow box. The analysis included only the dogs that entered each specific behavioral state (wakefulness *n* = 28, drowsiness *n* = 26, and NREM sleep *n* =24).

We calculated power, coherence, and Lempel Ziv complexity for every dog, except for one in which Fz, F3 and F4 electrodes were not analyzed because the dog removed those electrodes during the recording. Therefore, in this dog, power and complexity were analyzed only for Cz, and we could not perform coherence analysis. The power spectrum analysis is summarized in Figure 4. There were significant relationships with age, performance on the detour task and memory. For Fz, during drowsiness the power of slow oscillations (1–1.75 Hz) was negatively correlated with age and positively correlated with performance in the detour task. At each of the correlated frequencies, the strength of the correlation was higher for performance at detour task than for age. In addition to this, memory performance was correlated with the power of some frequency bands during REM sleep at Cz: dogs with higher memory scores had higher power of slow oscillations from 1 to 1.75 Hz, and lower power between 15.75 and 19 Hz (within beta frequency band). Also, at Fz, dogs with higher memory scores had lower power of higher oscillations, between 48 and 50 Hz (within gamma frequency band). Interhemispheric (F3–F4) coherence showed some correlations with age and cognitive function. As shown in Figure 5, during wakefulness, older dogs showed higher interhemispheric coherence for frequencies 6.5–13 Hz (within theta and alpha frequency bands). Dogs with higher CADES scores also had higher interhemispheric coherence at those frequencies, but the correlation was only significant between 10.5 and 12 Hz. Similarly, dogs with worse performance at the detour task showed higher interhemispheric coherence between 10.25 and 12 Hz. The strength of these correlations was higher for age than for CADES scores or performance at the detour task. We also found a negative correlation between performance in the sustained gaze task and interhemispheric coherence for slow oscillations up to 3 Hz (within delta frequency band). Furthermore, during REM sleep, older dogs had higher interhemispheric coherence for frequencies between 36.25 and 38 Hz.

**Figure 6:**
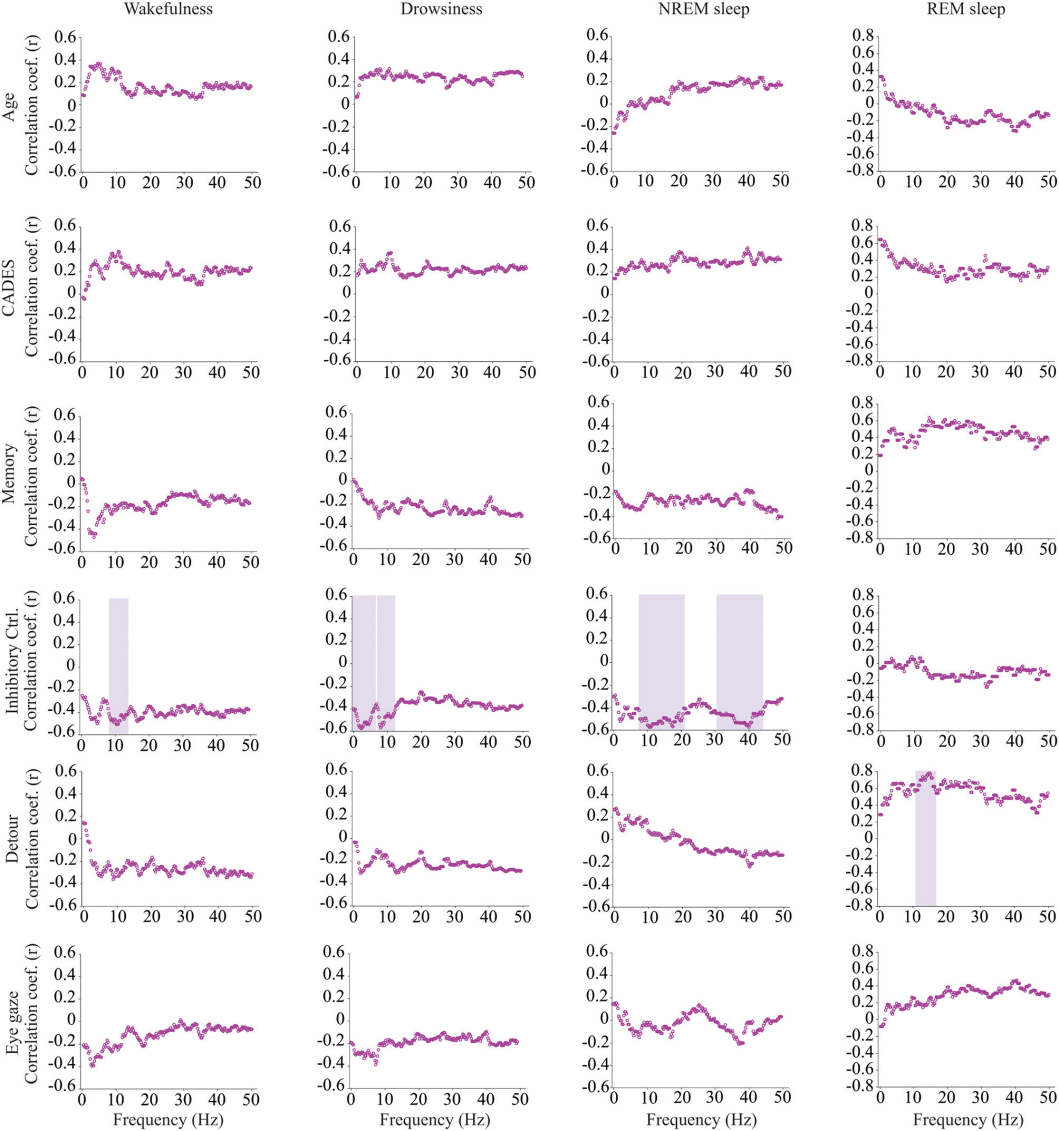
Correlations between EEG intrahemispheric (Fz-Cz) coherence and age, CADES score, or performance in cognitive tests. Spearman correlation coefficients are shown for each frequency bin. Frequencies with significant correlations are indicated with a purple shadow box. The analysis included only the dogs that entered each specific behavioral state (wakefulness *n* = 28, drowsiness *n* = 26, and NREM sleep *n* =24).

Intrahemispheric (Fz-Cz) coherence was correlated with cognitive function, but not with age (Figure 6). During wakefulness, dogs who performed better at the inhibitory control task had lower Fz-Cz coherence for the frequencies between 8 and 14.25 Hz. Similarly, during drowsiness, frequencies between 0.25–6.5 and 7–12.5 Hz were lower for dogs with better performance in the same task. During NREM sleep, there was also a negative correlation between coherence and inhibitory control scores, but for higher frequencies, between 7.75–20.75 and 30.25–44.25 Hz. During REM sleep, an association was found with performance on the detour task, dogs with better performance had higher coherence for frequencies between 10.75 and 16.5 Hz.

We evaluated the complexity of the EEG signal for the electrodes Fz and Cz and evaluated its correlation with age, CADES score, and performance in cognitive tests and we found a negative correlation between memory performance and complexity during REM sleep at Fz (Table 2). Age showed a positive correlation and performance in the detour task showed a negative correlation with complexity during REM sleep, but the significance was lost after multiple comparisons correction. Similarly, we observed that age tended to be positively correlated, while CADES, memory, and performance at the detour task tended to be negatively correlated with complexity during REM sleep at Cz. However, none of these remained significant after multiple comparisons correction (Table 3).

**Table 2:** Correlation between Lempel Ziv Complexity at Fz and age and cognitive function.

**Variable**	**By variable**	**Spearman (ρ)**	**Raw *p*-value**	***q*-value**
Age	LZC W	0.172	0.391	0.708
Age	LZC Drow.	0.291	0.158	0.398
Age	LZC NREM	0.268	0.215	0.433
Age	LZC REM	0.631	0.015^*^	0.124
CADES	LZC W	0.141	0.483	0.730
CADES	LZC Drow.	0.286	0.165	0.398
CADES	LZC NREM	0.269	0.215	0.433
CADES	LZC REM	0.438	0.117	0.398
Memory	LZC W	−0.036	0.866	0.950
Memory	LZC Drow.	0.025	0.908	0.954
Memory	LZC NREM	−0.178	0.440	0.708
Memory	LZC REM	−0.813	0.001^*^	**0.031** ^*^
Inhibitory control	LZC W	−0.078	0.698	0.887
Inhibitory control	LZC Drow.	−0.119	0.572	0.812
Inhibitory control	LZC NREM	0.002	0.992	0.998
Inhibitory control	LZC REM	−0.228	0.432	0.708
Detour	LZC W	−0.326	0.104	0.398
Detour	LZC Drow.	−0.343	0.100	0.398
Detour	LZC NREM	−0.342	0.119	0.398
Detour	LZC REM	−0.711	0.006^*^	0.078
Eye gaze	LZC W	0.049	0.806	0.946
Eye gaze	LZC Drow.	−0.087	0.679	0.887
Eye gaze	LZC NREM	−0.049	0.823	0.946
Eye gaze	LZC REM	−0.398	0.159	0.398

Asterisks indicate significant differences and bold text indicates significant differences after the false discovery rate Benjamini, Krieger, and Yekutieli procedure.

q-values represent the corrected p-values.

W, Wakefulness; Drow, Drowsiness; LZC, Lempel Ziv Complexity.

**Table 3:** Correlation between Lempel Ziv Complexity at Cz and age and cognitive function.

**Variable**	**By variable**	**Spearman ρ**	**Raw *p*-value**	***q*-value**
Age	LZC W	0.281	0.147	0.362
Age	LZC Drow.	0.337	0.092	0.362
Age	LZC NREM	0.304	0.148	0.362
Age	LZC REM	0.625	0.017^*^	0.362
CADES	LZC W	0.124	0.531	0.704
CADES	LZC Drow.	0.312	0.120	0.362
CADES	LZC NREM	0.275	0.193	0.362
CADES	LZC REM	0.572	0.033^*^	0.362
Memory	LZC W	−0.136	0.518	0.704
Memory	LZC Drow.	−0.056	0.1794	0.934
Memory	LZC NREM	0.010	0.966	1.000
Memory	LZC REM	−0.583	0.047^*^	0.362
Inhibitory control	LZC W	0.002	0.992	1.000
Inhibitory control	LZC Drow.	0.048	0.816	0.934
Inhibitory control	LZC NREM	0.055	0.798	0.934
Inhibitory control	LZC REM	−0.385	0.174	0.362
Detour	LZC W	−0.232	0.244	0.362
Detour	LZC Drow.	−0.343	0.093	0.362
Detour	LZC NREM	−0.315	0.143	0.362
Detour	LZC REM	−0.376	0.205	0.362
Eye gaze	LZC W	−0.242	0.214	0.362
Eye gaze	LZC Drow.	−0.244	0.230	0.362
Eye gaze	LZC NREM	−0.257	0.225	0.362
Eye gaze	LZC REM	−0.424	0.131	0.362

Asterisks indicate significant differences before the false discovery rate Benjamini, Krieger, and Yekutieli procedure.

q-values represent the corrected p-values.

W, Wakefulness; Drow, Drowsiness; LZC, Lempel Ziv Complexity.

## 4. Discussion

In this study we evaluated changes in sleep architecture objectively using polysomnography, the gold standard technique used to describe and quantify sleep (27, 28). We were able to obtain artifact-free EEG data using surface (cup) electrodes in elderly dogs while sleeping in the hospital setting. We demonstrated that dogs with higher dementia scores spend less time sleeping in both NREM and REM sleep in the afternoon than dogs with lower scores. This supports owner observations that disruptions of the sleep-wakefulness cycle characterize CCDS (11, 17). Similarly, humans with AD suffer from insomnia and lower sleep quality (60, 61), and a bidirectional relationship between sleep and disease has been proposed (13) in which amyloid burden in the brain disturbs sleep (62–64) while poor sleep promotes amyloid β deposition and impairs memory consolidation (65, 66).

We found that sleeping time was positively associated with the performance in a problem-solving detour task. Studies in humans have demonstrated that sleep plays a critical role in enhancing the skills needed to find novel strategies to solve problems (67, 68). One study has shown sleep dependent improvement in problem solving tasks, particularly those with higher difficulty (68). In dogs, Kis et al. have shown that sleep is associated with social learning skills (21). In the current study, longer sleep time was associated only with performance at the detour task and not with the other cognitive tasks. Similarly, a previous study on senior dogs (ages 7–14 years) testing the relationship between sleep spindles and cognition found that correlations were specific to, in that case, reversal learning only (69). Some human studies have shown that sleep deprivation can affect particular components of cognition differently (70–72). For example, Tucker et al. observed a larger effect of deprivation induced sleep loss in humans on non-executive rather than executive components of cognition, with working memory being less vulnerable than other domains (71). The main difference between human studies and our study is that they artificially deprive subjects to evaluate the effect of sleep deprivation, and in our study, reduction in sleep occurs naturally, likely due to aging and neurodegeneration. In addition, interindividual differences in the effects of sleep loss on cognition have been shown; some individuals can be more susceptible to attention impairment while others show working memory reduction (73). In dogs, the specific domains of cognition that are impaired by sleep loss are vastly understudied, with a sole paper suggesting that disturbance of NREM sleep affects dogs' socio-cognitive processing (74), but without reporting on the potential interindividual differences. The more challenging nature of the detour task we used, encompassing the interaction of multiple cognitive domains (18) may make this test more susceptible to the effect of sleep loss than other tests. This task requires cognitive stability (the ability to focus) and cognitive flexibility (the ability to adapt behavioral actions due to a change in demands) (75). We have previously shown that the detour task, together with inhibitory control and sustained gaze, is better able to discriminate between dogs with and without cognitive dysfunction than other tests such as the working memory task (18).

### 4.1. Power and age

Quantification of the EEG signal using power spectrum, coherence and complexity in different behavioral states revealed associations with age and cognitive function (25). As previously mentioned, power spectrum calculates the relative contribution of the frequency components of a signal, and it is a measure of local synchronization. During drowsiness, power of slow oscillations was lower for older dogs and for dogs with worse performance at the detour task. These oscillations are within the delta range and are characteristic of states of diminished levels of consciousness such as NREM sleep and anesthesia (76, 77). Our findings are consistent with the observation in humans (78), rats (79), cats (80), and dogs (28), that delta power declines with aging during NREM sleep, and this is considered a hallmark of reduction in sleep depth (81–83). In our study, the reduction in delta power with age was observed during both drowsiness and NREM sleep, however it only reached statistical significance for drowsiness. Of note, these associations were only observed in the Fz EEG channel, not in the Cz. In humans, it has been observed that frontal brain areas are more sensitive to aging and to response to sleep loss, and while in young subjects there is a predominance of delta waves in the frontal cortex, this predominance is lost with aging (84).

### 4.2. Power and memory

We identified associations between power and memory scores but not age during REM sleep, the period in which memory consolidation occurs. By contrast, Kis et al. (28) found an age-associated reduction in delta and an increase in alpha and beta power in dogs. However, they studied dogs from 1 to 8 years of age, without including senior or geriatric dogs. Performance at the memory task was positively correlated with delta power and negatively correlated with power at higher frequencies (beta and gamma). The biological relevance of this finding should be further evaluated in future studies and include dogs across a wider range of ages together in one study. While REM sleep is usually treated as a homogenous state, two different phases have been recognized, tonic and phasic REM sleep. These phases have different roles in information processing and memory consolidation and differ in the power of different oscillations, with higher power of alpha and beta bands during the tonic phase and higher gamma power on the phasic phase (85–87). Studies in dogs are needed to characterize the electroencephalographic features of each REM phase and whether they are associated with cognition and cognitive function.

### 4.3. Interhemispheric coherence and age

Coherence analyses are a measure of functional coupling between two distant cortical areas. Higher interhemispheric coherence within theta and alpha frequency bands was found in older dogs during wakefulness. This result was unexpected since in people, coherence in theta and alpha band decreases with aging (88, 89). However, those human studies included a wider range of ages, including young, middle age and senior adults, while in our study, we evaluated only senior and geriatric dogs. Our current findings concur with a previous study we performed in a separate cohort of dogs in which we found that dogs at risk of developing CCDS had higher interhemispheric coherence values than normal dogs or dogs with CCDS (41). It is important to note that those dogs at risk were also older than the normal dogs, and differences between groups could also be age-related.

During REM sleep we observed a positive correlation between gamma interhemispheric coherence and age. In the normal individual, absence of gamma coherence characterizes REM sleep (90, 91), and gamma coherence shows its peak during wakefulness. Therefore, higher gamma coherence during REM sleep could be caused by shallower sleep states and frequent arousals and could suggest an age-related reduction in quality sleep in these dogs.

### 4.4. Interhemispheric coherence and cognition

There was a positive correlation between interhemispheric coherence and CADES score and a negative correlation with performance at the detour task during wakefulness. This was true for frequencies between 10.5 and 12 Hz, some of the same frequencies for which coherence was positively correlated with age. For these frequencies, the strength of the correlations was higher for age than for CADES or performance at the detour task. We have previously demonstrated that cognitive performance decreases with age (18), and therefore, it is possible that age is the main factor driving these coherence differences.

During wakefulness, delta interhemispheric coherence (1–3 Hz) showed a negative correlation with performance at the sustained gaze task, a test that measures sustained attention. Dogs with shorter attention during the task had higher coherence. This mirrors studies in people with dementia that have shown an increase in delta coherence in comparison with healthy age-matched controls (40, 92). In addition to this, patients with attention deficit-hyperactivity disorders have elevated delta coherence in the frontal area of the brain (93).

In our previous study we found that dogs with higher dementia scores had lower interhemispheric coherence in the gamma frequency band during wakefulness, but those results were not replicated in this study. One of the main reasons could be that in our previous study we used needle electrodes that have a much smaller recording surface than the cup electrodes. In fact, Musteata et al. found that values of interhemispheric coherence in dogs are dependent on the type of electrode used (94). A limitation of coherence analyses is that coherence values can be overestimated due to volume conduction in which the conductive properties of the brain, cerebrospinal fluid, skull, and scalp (and the conductive gel used during the recording), cause the neural source information to diffuse before reaching the electrodes (95, 96). Therefore, coherence measurements with cup electrodes might not be completely reliable in dogs, and future studies should evaluate more advances measures of functional connectivity that are able to overcome the volume conduction problem such as the phase lag index (95, 97).

### 4.5. Intrahemispheric coherence and cognition

Intrahemispheric coherence showed associations with performance in cognitive tasks but not with age or CADES scores. Higher coherence in different frequency bands during wakefulness, drowsiness and NREM sleep were associated with worse performance at the inhibitory control task. In humans with AD, Sankari et al. found an increase in delta, theta, and alpha intrahemispheric coherence during wakefulness (98). Finally, during REM sleep, dogs with better performance at the detour task showed higher intrahemispheric coherence within the alpha and sigma frequency bands. REM sleep in humans is characterized by the occurrence of alpha bursts with high occipito-frontal connectivity (99). It has been proposed that these alpha bursts may contribute to higher information processing during this state, and to the incorporation of external stimuli to dreams (99, 100).

### 4.6. Complexity and memory

Lempel Ziv Complexity evaluates the randomness of the EEG signal. We did not find any effect of aging on EEG complexity in any of the behavioral states evaluated. Regarding its association with cognitive status, dogs with better performance in memory tasks had a lower EEG complexity during REM sleep. Similarly, dogs with better performance in the detour task had also lower REM sleep complexity, but this result did not achieve statistical significance after multiple comparisons correction. It has been shown that complexity has its maximum value during wakefulness, is reduced during NREM sleep and increases to intermediate values during REM sleep (101, 102). Therefore, higher levels of complexity during REM in dogs with worse cognitive performance can be an indication of increased arousal, or shallower REM sleep.

Together these EEG findings demonstrate that dogs with cognitive impairment spend less time sleeping and show EEG features of shallower sleep than dogs without cognitive impairment. Additionally, changes in power and coherence of specific frequency bands are associated with age or performance at some cognitive tests in aging dogs.

### 4.7. Limitations

Polysomnographic recordings in this study were completed during afternoon sleep and thus might not reflect the sleep architecture of dogs at nighttime. While there is evidence that at nighttime, dogs sleep more and spend less time in drowsiness and awake after first drowsiness (103), there are no data showing whether day vs. night sleep patterns are differentially affected by age (which would be a limitation to the present study). Thus, we do not know if and to what extent the present results would change if replicated during night-time recordings. Recordings during nighttime require considerably more effort from both the clinical staff and the participating owners, thus afternoon recordings are much more feasible for veterinary settings. Wireless polysomnographic equipment with bandages, Elizabethan collars and or special EEG caps designed to assure that electrodes stay in place (104) were not available for this study. In addition to this, while it has been demonstrated that dogs usually take naps around noon (5, 53), there is individual variability, and for some dogs the time chosen for recordings might not be their preferred nap time. However, the results observed in this study are in line with the sleep difficulties experienced by dogs with CCDS at night, since, as expected (9, 11), dogs with higher CADES scores spent less time sleeping. Future studies could compare these afternoon polysomnographic recordings with easier evaluations of activity at night time such as activity monitors (53) or sleep questionnaires (105). In addition to this, we have already demonstrated that cognitive function decreases with aging (18), and therefore, it is challenging to determine the relative contribution of age and cognitive impairment on changes in the sleep-wakefulness cycle and the qEEG features.

## 5. Conclusion

In conclusion, this study evaluates for the first time the correlation between sleep architecture and cognitive performance in aging dogs using polysomnography, the gold standard technique for sleep evaluation. We have demonstrated that dogs with higher CADES scores spent less time in both NREM and REM sleep. Additionally, when evaluating different domains of cognition, problem solving abilities showed a positive correlation with time spent in NREM and REM sleep and a negative correlation with time awake. Finally, we showed that quantitative electroencephalographic analyses during different behavioral states can demonstrate differences in dogs associated with age or cognitive performance which should be further evaluated for its potential clinical use to monitor the progression of CCDS.

## Data availability statement

The raw data supporting the conclusions of this article will be made available by the authors, without undue reservation.

## Ethics statement

The animal study was reviewed and approved by Institutional Animal Care and Use Committee NCSU. Written informed consent was obtained from the owners for the participation of their animals in this study.

## Author contributions

AM and NJO were responsible for the design, analysis, and primary writing of the manuscript for this study. AK and MEG participated in the design. AM, CL, and MK participated in the data acquisition. DM and MC participated in the analysis of the data. All authors participated in editing and review of the manuscript. All authors contributed to the article and approved the submitted version.
